# The impact of an AIPER on health behavior improvement among sedentary adults: a longitudinal extension of the TAM

**DOI:** 10.3389/fpsyg.2025.1738594

**Published:** 2026-01-20

**Authors:** Zhaoyu Liu, Soohyun Kim

**Affiliations:** Department of Sport and Healthcare, Namseoul University, Cheonan, Republic of Korea

**Keywords:** AIPER, health behavior improvement, health self-efficacy, sedentary individuals, TAM

## Abstract

**Background:**

Sedentary behavior has become a major public health concern and is closely associated with various unhealthy behaviors and chronic diseases. Artificial intelligence shows promise for promoting health behavior change through personalized exercise interventions.

**Objective:**

To examine, over a 6-month period, the effect of an AI-based Personalized Exercise Recommendation System (AIPER) on Health Behavior Improvement (HBI) among sedentary adults and to analyze its psychological mechanisms.

**Methods:**

A two-wave survey (T1 and T2, 6 months apart) was conducted with 492 sedentary participants. Measures covered TAM-related behavioral variables—Perceived Ease of Use (PEOU), Perceived Usefulness (PU), Attitude Toward Use (ATU), Behavioral Intention (BI), and System Use (SU)—as well as Health Self-Efficacy (HSE), Health Behavior Improvement (HBI), and demographics. Factor analyses, correlation analyses, and structural equation modeling were performed using SPSS 23.0 and Amos 23.0.

**Results:**

AIPER significantly promoted HBI among sedentary adults. Chain mediation effects were identified, whereby PEOU and PU influenced ATU and BI, and together with HSE indirectly affected SU, ultimately improving HBI.

**Conclusion:**

AIPER can increase SU and indirectly improve HBI in sedentary populations. It is recommended that government agencies, enterprises, and universities/research institutes implement AI health-management systems, formulate individualized and scientifically grounded behavior-change plans, enhance the health behaviors of sedentary groups, and advance personalized, evidence-based, and sustainable public health promotion.

## 1 Background

Sedentary behavior has been identified by the World Health Organization as a major contributor to the global health crisis. Prolonged sitting is closely associated with cardiovascular disease, metabolic syndrome, and mental health problems (Bull et al., 2020). With the increasing shift of work and study to online environments, together with the proliferation of digital entertainment, sedentary behavior has become more prevalent, insidious, and socially normalized, gradually giving rise to a “static lifestyle” (Goyal and Rakhra, 2024). Beyond physical health risks, recent research has further highlighted that sedentary behavior also carries profound implications for brain health, cognitive functioning, and emotional wellbeing. Zou and colleagues’ recent work systematically synthesizes evidence demonstrating that prolonged sedentary patterns are related to poorer cognitive performance, alterations in brain structure and function, and an elevated risk of adverse neuropsychological outcomes, while also raising concerns regarding emotional and affective consequences of sedentary lifestyles (Zou et al., 2024). At the same time, their studies emphasize both the methodological challenges in measuring sedentary behavior and the emerging potential of digital and intelligent technologies to better monitor and intervene in sedentary lifestyles.

With advances in artificial intelligence for health management and exercise intervention, AI systems are playing an increasingly important role in providing personalized exercise support. By integrating users’ physiological indicators, daily behavioral patterns, and health goals, AI-based personalized exercise recommendation systems (AIPERs) are capable of sensing individual differences in real time and dynamically generating tailored exercise prescriptions. Compared with traditional experience-based guidance or standardized intervention protocols, this data-driven approach markedly improves the scientific rigor, targeting accuracy, and implementation efficiency of exercise prescriptions (Chin et al., 2022).

However, the intelligence of a technology does not automatically guarantee intervention effectiveness. Whether an AIPER is continuously adopted and stably used depends critically on users’ perceived experience (Ibrahim et al., 2025). In populations where exercise habits are not yet established and health awareness may be relatively weak—such as sedentary individuals—the system’s usability, feedback modality, and personalization level may all play key roles in shaping whether the technology is adopted and whether adoption translates into actual health behavior change. Users’ perceptions of operational convenience and their evaluations of whether the system helps achieve health goals—Perceived Ease of Use (PEOU) and Perceived Usefulness (PU)—constitute core cognitive antecedents of Behavioral Intention (BI) (Davis, 1989). Meanwhile, psychological variables formed during system interaction, such as Attitude Toward Use (ATU) and Health Self-Efficacy (HSE), may further influence subsequent System Use (SU) through chained psychological pathways. In particular, when interface interaction is intuitive, task execution requires limited cognitive effort, and feedback is timely and clear, users are more likely to develop confidence, increase perceived capability, and enhance their willingness to persist in health-related actions (Bandura, 1997).

Building upon this neurocognitive and psychological understanding of sedentary behavior, research attention needs to move beyond the question of whether AI systems are adopted toward whether their use can truly translate into meaningful and sustained improvements in health behavior. However, existing technology acceptance research has largely concentrated on the intention-formation stage, paying relatively limited attention to whether post-adoption system use leads to positive and lasting health behavior outcomes. In the intervention context of AI-based personalized exercise recommendations, the psychological and behavioral change mechanisms following system adoption remain insufficiently verified. Moreover, most current studies rely on cross-sectional designs, which are unable to capture the dynamic evolution of users’ perceptions, psychological processes, and behavioral performance over time, nor can they clarify the temporal and causal mechanisms underlying such change. Longitudinal research, by tracking the same cohort over time, is therefore better positioned to reveal the sustained pathways through which AI systems influence Health Behavior Improvement (HBI).

Accordingly, this study employed a two-wave longitudinal design, following sedentary individuals over six months to observe time-varying changes in user perceptions, psychological processes, system use, and health behavior outcomes. Building on the Technology Acceptance Model (TAM), this study constructed an extended explanatory framework incorporating affective attitudes and motivational mechanisms to analyze the temporal relationships among perceptions, intentions, self-efficacy, system engagement, and health behavior. The aim was to elucidate how an AIPER influences sustained system use and HBI through users’ cognitive evaluations and psychological regulation mechanisms. By doing so, this research not only extends TAM into the temporal dimension and into the domain of sedentary behavior and health promotion, but also provides longitudinal empirical evidence and theoretical grounding for the application of AI-based personalized exercise systems in promoting healthier, more active lifestyles among sedentary populations.

## 2 Literature review and research hypotheses

### 2.1 From AIPER → PEOU → PU → ATU → BI → system use

In this study, the AIPER does not refer to a generic intelligent exercise recommendation tool; rather, it denotes an AI-based personalized exercise recommendation system that dynamically learns from individual differences and continuously optimizes recommendations through real-time feedback. Its personalization logic mainly operates across three layers: initial user profiling based on basic characteristics (e.g., age, gender, health status, and exercise experience), continuous monitoring of behavioral indicators such as exercise frequency, duration, completion rate, heart rate, or perceived exertion, and adaptive optimization through a dynamic “assessment–recommendation–feedback–re-optimization” loop. In practical application scenarios, users input or synchronize their health and exercise data via mobile applications or smart devices, through which the system generates individualized recommendations regarding exercise type, intensity, frequency, and adjustment prompts, along with motivational feedback and corrective guidance during system use. Thus, AIPER functions as a companion-type, learning-oriented, and interactive support system that reduces information burden and technological effort, enhances perceived ease of use, and further facilitates positive attitude formation and continued usage intention (Venkatesh and Davis, 2000; Davis, 1989; Lee et al., 2003).

Within the Technology Acceptance Model (TAM), perceived ease of use (PEOU) influences perceived usefulness (PU), while both jointly shape individuals’ attitude toward use (ATU), which subsequently determines behavioral intention (BI) and actual system use (SU) (Davis, 1989; Venkatesh and Davis, 2000; Lee et al., 2003). Consistent with Ajzen’s (1991) theory of planned behavior, intention serves as the most direct predictor of actual behavior. In digital health contexts, this chain relationship has been repeatedly validated, suggesting that when individuals perceive a system as easy to use and useful, they form favorable attitudes, subsequently developing intention and behavioral engagement.

### 2.2 From AIPER → PEOU → PU → BI → system use

Based on the general TAM relationships described in Section 2.1, this pathway emphasizes a cognition-dominant mechanism in which users’ ease-of-use perception and usefulness evaluation directly shape behavioral intention, even in the absence of explicit affective responses. When an AI health system offers intuitive interfaces, efficient functions, and low learning costs, users may focus primarily on whether the system is genuinely beneficial, forming BI without relying heavily on emotional attitude formation. Empirical evidence indicates that PEOU can significantly influence BI through PU, even when ATU is not explicitly modeled (Venkatesh and Bala, 2008; Zhao et al., 2018). This pathway is particularly applicable to task-driven users—such as those engaged in fitness planning, rehabilitation monitoring, or chronic-condition management—who primarily evaluate AIPER as a performance-oriented tool. Once convinced of its instrumental effectiveness, they tend to form firm BI that further translates into stable SU (Holden and Karsh, 2010).

### 2.3 From AIPER → PU → ATU → BI → system use

This pathway highlights a functionality-centered route in which users’ cognitive judgments of system usefulness play a central role in shaping emotional attitude and behavioral decisions. Personalized exercise recommendations provide goal-congruent, high-value support, enabling users to recognize AIPER as an effective facilitator of health outcomes, thereby enhancing PU. Compared with pathways prioritizing PEOU, this mechanism focuses on users’ confidence in whether the system meaningfully supports goal attainment. When PU is strong, users tend to develop positive emotional responses—such as trust, confidence, or satisfaction—which form the psychological foundation of system use (Bhattacherjee and Premkumar, 2004). Consequently, stronger ATU leads to higher BI and promotes continuous SU. This pathway is particularly salient in performance-oriented contexts, emphasizing functional cognition as a core motivational driver of system use (Martins et al., 2014).

### 2.4 From AIPER → PU → BI → system use

This pathway further shortens the affective mediation chain, illustrating how rational judgments of system utility alone may directly shape BI and sustained usage behavior. When AIPER provides accurate, individualized, and outcome-relevant guidance, users can rely primarily on instrumental evaluation to decide whether to continue using the system. In repetitive health management scenarios—such as exercise monitoring, rehabilitation planning, or chronic-condition tracking—users tend to evaluate technological tools mainly on outcome effectiveness rather than emotional experience (Sun and Zhang, 2006). Accordingly, PU serves as a dominant driver; once users perceive substantial functional value, they readily convert such cognition into BI, which then stabilizes into consistent SU (Gefen and Straub, 2000; Wixom and Todd, 2005).

### 2.5 From AIPER → PEOU → ATU → BI → system use

Unlike the pathways emphasizing PU as the central mediator, this mechanism underscores the affective role derived from perceptual experience. In AIPER contexts, users may not initially assess long-term health outcomes; instead, intuitive perceptions of interface friendliness, interaction fluency, and operational simplicity can quickly stimulate positive attitudes. When users feel that the system is easy to navigate, requires minimal effort, and supports smooth interaction, they are likely to experience enjoyment, comfort, and trust, forming favorable ATU (Mun and Hwang, 2003). In such cases, PEOU acts not merely as a cognitive determinant of PU but as a direct emotional stimulus shaping attitudes. Prior digital health research confirms that enjoyable, low-effort user experiences significantly foster BI and long-term adherence (Zhang et al., 2018; Holden and Karsh, 2010). Thus, PEOU in this pathway functions as both a technical and psychological motivator driving system use.

### 2.6 From AIPER → PEOU → HSE → system use

In this pathway, perceived ease of use functions not merely as an experiential variable that reduces technological barriers, but as a psychological catalyst that strengthens individuals’ belief in their ability to regulate health behaviors. When the AIPER provides intuitive interaction, clear behavioral guidance, and timely feedback, users gradually develop a sense of control over the exercise management process, thereby enhancing Health Self-Efficacy (HSE)—that is, the belief that one is capable of initiating, sustaining, and regulating health-related actions. This capability belief further promotes more stable and persistent behavioral engagement under system guidance (Yamin et al., 2011).

Unlike the traditional TAM logic, which primarily explains technology adoption through a linear “perception–attitude–intention” pathway, this study conceptualizes HSE as an independent and critical motivational mediator that bridges technological perception and sustained behavioral maintenance. In AI-based personalized health intervention contexts, users not only need to decide whether to use the system but also whether they believe they are capable of continuously acting upon the system’s recommendations. Therefore, high perceived ease of use influences not only attitudes and intentions but also strengthens users’ capability beliefs and sense of control over health goals, which is particularly crucial during the early stages of behavior establishment, such as the transition from sedentary to active lifestyles. Existing studies have also demonstrated that enhanced self-efficacy significantly improves long-term adherence to health technologies and promotes sustained participation (Bailey et al., 2015).

Accordingly, this pathway does not merely reflect a simple cognitive chain, but rather a cognitive–motivational mechanism: perceived ease of use facilitates the formation of efficacy beliefs; efficacy beliefs drive system use; and sustained system use provides the foundation for subsequent health behavior improvement. In the current hybrid online–offline health management environment, systems that effectively enhance individuals’ sense of capability are more likely to foster early habit formation, behavioral maintenance, and long-term improvement in health outcomes (Bol et al., 2018).

### 2.7 System use → health behavior improvement

In this study, System Use (SU) and Health Behavior Improvement (HBI) are conceptualized as two theoretically distinct constructs rather than as components on a single behavioral continuum. SU reflects a technology-engagement behavior, referring to the extent to which individuals log in, interact with, and utilize the AIPER system. By contrast, HBI represents a health-behavior outcome, indicating whether users have genuinely internalized the system’s recommendations and transformed them into sustained positive changes in exercise frequency, regularity, persistence, and activity intensity.

Conceptually, SU is regarded as a necessary but insufficient condition for HBI. Users must first continuously interact with the system in order to receive guidance, adaptive feedback, and personalized exercise recommendations; however, merely “using the system” does not automatically ensure meaningful behavioral transformation. Users may demonstrate shallow use, intermittent participation, or compliance-driven engagement without psychological commitment to behavior change. Therefore, SU is treated as a behavioral gateway that opens the pathway toward change, whereas HBI captures a qualitatively higher-order transformation, involving behavioral execution, internalization, and long-term maintenance.

While TAM and its extensions have solidly explained how attitudes and intentions predict technology use (Holden and Karsh, 2010), much less attention has been paid to the post-adoption stage, particularly whether system use leads to tangible and sustainable health outcomes. In AI-driven personalized exercise contexts, continued SU is essential for reinforcing habits through ongoing feedback loops, motivational reinforcement, and adaptive guidance; however, the transition from use to improvement is neither automatic nor guaranteed (Bhattacherjee and Premkumar, 2004). Accordingly, SU in this study is reconceptualized as a mediating mechanism that links psychological and technological processes to real behavioral outcomes, thereby extending TAM from explaining “why people use systems” to explaining “how technology-enabled engagement evolves into sustained behavior change.”

Empirical evidence further supports this conceptualization. Continuous use of AI-based personalized exercise systems has been shown to enhance adherence, promote active engagement with physical activity, reduce sedentary behavior, and strengthen the regularity and persistence of exercise participation (Wang et al., 2020). These changes collectively constitute HBI in this study and represent the meaningful transformation that occurs beyond mere system adoption.

**FIGURE 1:**
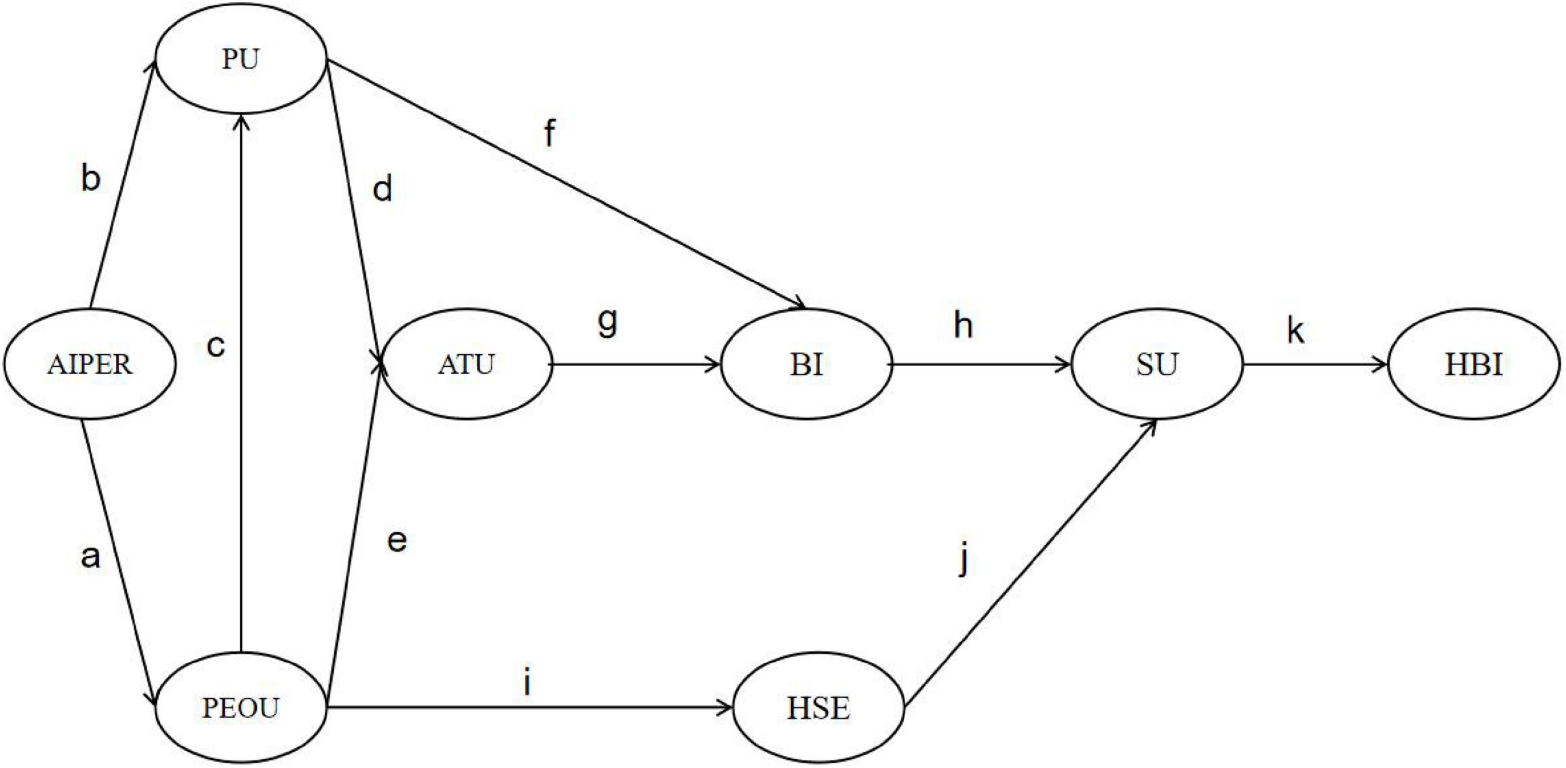
Research model. T1: AIPER; PEOU; PU; ATU; BI; SU; HSE. T2: HBI = Health Behavior Improvement. Effect “acdghk”: AIPER → PEOU → PU → ATU → BI → SU → HBI; Effect “acfhk”: AIPER → PEOU → PU → BI → SU → HBI; Effect “bdghk”: AIPER → PU → ATU → BI → SU → HBI; Effect “bfhk”: AIPER → PU → BI → SU → HBI; Effect “aeghk”: AIPER

Moreover, if TAM is interpreted solely through a linear technological pathway, it becomes difficult to uncover the differentiated psychological processes underlying “why users adopt,” “whether they are willing to continue using,” and “whether they are capable of persistently acting upon system guidance.” Therefore, this study does not simply stack multiple mediation chains. Instead, the model is theoretically organized into three higher-order psychological mechanisms: (1) Cognitive Pathways, driven by continuous information processing involving PEOU, PU, ATU, and BI; (2) Affective Pathways, emphasizing emotional evaluation embedded in attitude formation; and (3) Motivational Pathways, highlighting the empowering role of Health Self-Efficacy (HSE) in sustaining behavior. The six chain mediation paths (H1–H6) illustrated in Figure 1 should therefore be understood as concrete instantiations of these higher-order mechanisms rather than redundant incremental extensions of traditional TAM.

Consistent with the above theoretical categorization, the hypotheses are grouped according to the three higher-order mechanisms. H1–H4 belong to the cognitive information-processing mechanism, H5 reflects the affective evaluation mechanism, and H6 represents the motivational empowerment mechanism.

*H1:*    In the process through which the AIPER influences HBI, PEOU, PU, ATU, BI, and SU jointly exert a significant chained mediating effect.
*H2:*    In the process through which AIPER influences HBI, PEOU, PU, BI, and SU jointly exert a significant chained mediating effect.
*H3:*    In the process through which AIPER influences HBI, PU, ATU, BI, and SU jointly exert a significant chained mediating effect.
*H4:*    In the process through which AIPER influences HBI, PU, BI, and SU jointly exert a significant chained mediating effect.
*H5:*    In the process through which AIPER influences HBI, PEOU, PU, ATU, BI, and SU jointly exert a significant chained mediating effect.
*H6:*    In the process through which AIPER influences HBI, PEOU, HSE, and SU jointly exert a significant chained mediating effect.

In this study, the longitudinal design is not merely a methodological enhancement but carries explicit theoretical significance. Time is conceptualized as a critical dimension through which psychological and behavioral mechanisms unfold: as users continuously interact with the AIPER system, they undergo a dynamic process from learning and adaptation, to familiarity and trust building, and ultimately to behavioral habit formation and stabilization. This implies that the relationships among perceptions, intentions, system use, and health behavior improvement are not formed instantaneously, but are progressively strengthened and structurally consolidated over time. Therefore, the purpose of adopting a longitudinal design in this study is to examine the time-ordered causal logic—specifically, whether intention translates into sustained system use, and whether sustained use further leads to stable and maintainable health behavior improvement—rather than merely demonstrating temporal associations. This provides theoretically meaningful evidence for understanding the dynamic evolution of TAM within health behavior contexts.

## 3 Materials and methods

### 3.1 Participants

A combination of convenience sampling and snowball sampling was employed to recruit participants characterized by sedentary lifestyles. The sample primarily consisted of individuals recruited through universities, enterprises, and community health centers. Prior to data collection, the research team provided participants with a detailed explanation of the study’s purpose, follow-up schedule, and data collection procedures, and obtained their informed consent.

**FIGURE 2:**
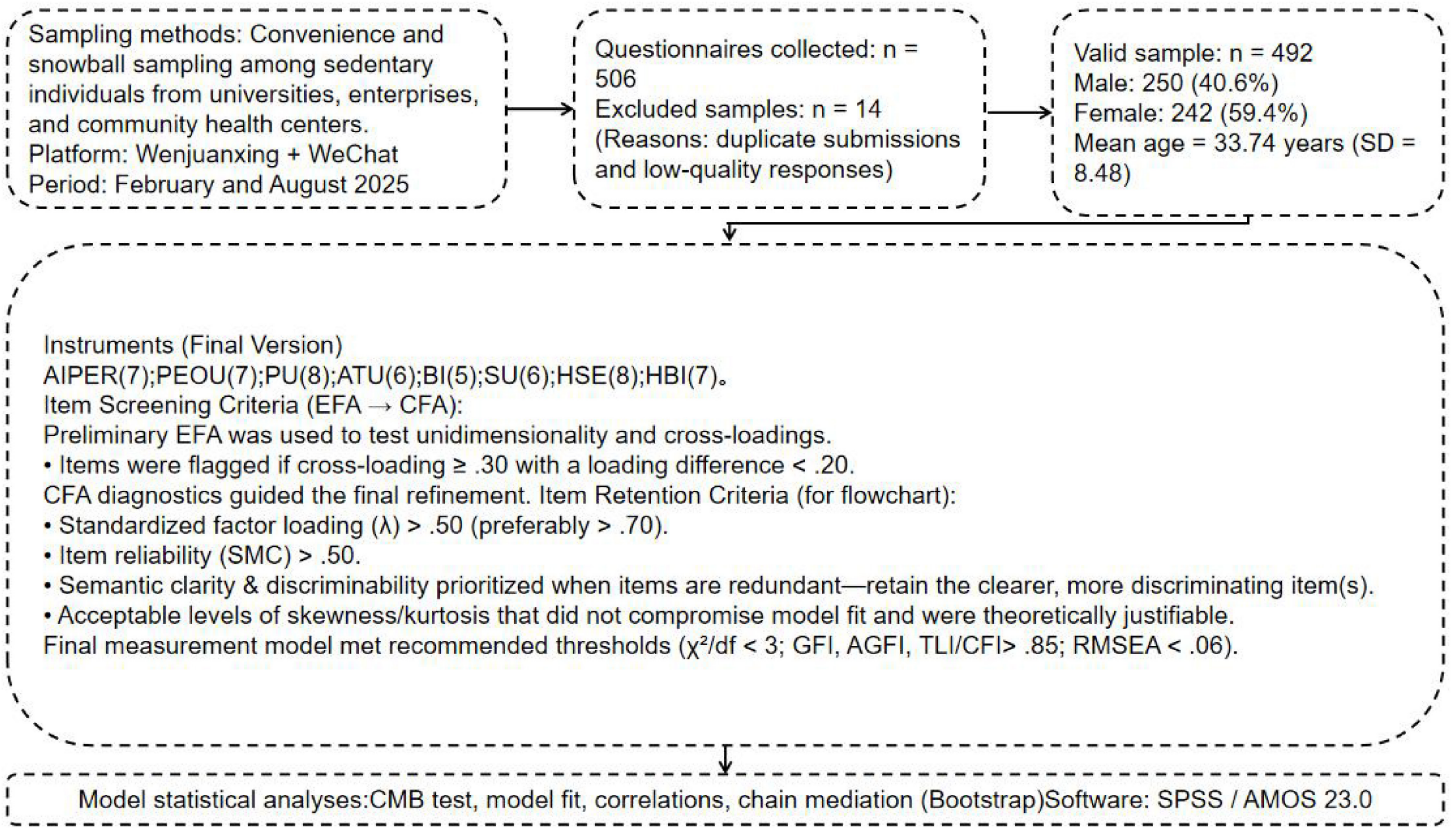
Research flowchart.

The survey was administered via the Questionnaire Star online platform and distributed through WeChat channels using an invitation and sharing mechanism to enhance participant diversity. Respondents completed the questionnaire anonymously, and small incentives were provided to encourage participation and ensure data reliability. This approach aimed to improve both the stability and validity of the data collected. Figure 2 illustrates the research procedure.

#### 3.1.1 Sample size estimation

A Structural Equation Modeling (SEM) approach was employed for empirical testing. According to established guidelines, the recommended sample size for SEM is typically 5–10 times the number of observed variables (Hair et al., 2019). With 54 items in this study’s questionnaire, the estimated sample size ranged from 270 to 540 (54 × 5 ≤ n ≤ 54 × 10), ensuring sufficient statistical power and model stability. Given the complex, chained structure of the model, Kline (2023) suggested that samples smaller than 400 may not be adequate for reliable path estimation; therefore, the minimum target sample size was set at 400.

#### 3.1.2 Survey waves and timeline

A two-wave longitudinal design was adopted. The first wave (T1) took place in February 2025, and the second wave (T2) in August 2025, with a 6-month interval. This interval was intended to reduce common method bias and to track intra-individual changes. Each participant was assigned a unique anonymous code at T1 to enable accurate matching at T2.

#### 3.1.3 Sample attrition and retention

In the first wave (T1), 531 valid questionnaires were collected, while 506 valid responses were obtained in the second wave (T2). The matching retention rate between T1 and T2 was 93.5%. After excluding invalid or incomplete cases (e.g., patterned responses, excessive missing data, or inconsistent answers), 492 valid paired responses were retained for analysis, yielding an effective rate of 92.6%. The data collection was completed on August 26, 2025.

#### 3.1.4 Participant characteristics

The final 492 valid respondents were all adults (≥ 18 years old) characterized by long-term sedentary behavior. The average age was 33.74 years (SD = 8.48). Among them, 250 (50.81%) were male and 242 (49.19%) were female. Detailed demographic information is presented in Table 1.

**TABLE 1:** Basic demographic information.

Variable	Category	N/%	Variable	Category	N/%
Gender Daily sitting duration	Male	250/50.81%	Preferred post-sedentary exercise type	Breaking sedentary behavior	230/46.75%
	Female	242/49.19%		Low-to-moderate aerobics	71/14.43%
	≤ 6 h	107/21.75%		Muscle strength and resistance	112/22.76%
	6–8 h	232/47.15%		Stretching and relaxation	43/8.74%
	≥ 8 h	153/31.10%		Body shaping	36/7.32%
Exercise experience Education level	< 3 years	96/19.51%	Commonly used exercise recommendation tool	Wearable device	161/32.72%
	3–5 years	249/50.61%		Large-screen display system	99/20.13%
	≥ 5 years	147/29.88%		Mobile exercise app	142/28.86%
	Below bachelor’s	76/15.45%		AI-based virtual coaching	47/9.55%
	Bachelor’s or above	416/84.55%		Psychological regulation system	43/8.74%

### 3.2 Measurement instruments

T1: AIPER scale (7 items) was adapted from Pu et al. (2011), which focuses on system recommendation satisfaction, and from Komiak and Benbasat (2006), who conceptualized trust and personalization in AI systems. PEOU (7 items) and PU (8 items) were derived from the TAM developed by Davis (1989) and contextualized for AI-based exercise recommendations. ATU (6 items) was based on Bhattacherjee and Premkumar (2004), incorporating satisfaction and extended use intention constructs. BI (5 items) was revised and validated following Rosli et al. (2023) and Bhattacherjee (2001) under the Expectation-Confirmation Model framework. HSE (8 items) was modified from Smith et al. (1995) Perceived Health Competence Scale. SU (6 items) was developed with reference to Burton-Jones and Straub (2006) Deep Structure Usage and Doll and Torkzadeh (1998) system usage measures. T2: HBI scale (7 items) was adapted from Sechrist et al. (1987) Exercise Benefits/Barriers Scale and Walker et al. (1987) Health-Promoting Lifestyle Profile. Items were translated and refined to fit the research context, ensuring semantic and cultural equivalence for Chinese respondents.

## 4 Data analysis

All statistical analyses were conducted using IBM SPSS Statistics 23.0 and Amos 23.0 for Windows. All scale items were evaluated based on a 7-point Likert scale, with a significance level set at 0.05.

First, to minimize potential systematic errors, common method bias was examined to ensure the reliability of the measurement data. Next, Confirmatory Factor Analysis (CFA) was conducted to optimize item selection and validate the structural validity of the scales, confirming the rationality and stability of each measurement instrument.

On this basis, descriptive statistics were performed, calculating means, standard deviations, and frequency distributions for each variable to describe the basic characteristics of the sample.

After descriptive analysis, the study proceeded to correlation analysis to examine the interrelationships among variables. Finally, to test the chained mediation effects, the study employed Amos (Estimation: Maximum Likelihood) and Bootstrap resampling (*n* = 2,000) to construct and verify the hypothesized model’s path coefficients and indirect effects.

This analytical procedure examined how AIPER, PU, PEOU, ATU, BI, HSE, and SU jointly influenced HBI, thereby revealing the mediating mechanisms among these constructs.

### 4.1 Common method bias and model fit examination

To reduce the potential impact of systematic bias from a single data source, the questionnaire design incorporated several procedural controls, including confidentiality assurances, randomization of item order, and balanced wording of items. During statistical analysis, both single-factor and multi-factor CFA were conducted to examine the presence of common method bias (Podsakoff et al., 2003).

In the single-factor model, the fit indices were χ^2^ = 12,690.376, *df* = 1377, χ^2^/*df* = 9.216, GFI = 0.346, AGFI = 0.294, TLI = 0.402, CFI = 0.424, and RMSEA = 0.129. For the multi-factor model, the fit indices were χ^2^ = 1758.092, *df* = 1366, χ^2^/*df* = 1.287, GFI = 0.887, AGFI = 0.878, TLI = 0.979, CFI = 0.980, and RMSEA = 0.024. The markedly better fit of the multi-factor model indicates that the dataset is not affected by serious common method bias.

As shown in Table 2, the overall model fit met or exceeded recognized standards (Hair, 2009; Hu and Bentler, 1999). Although GFI and AGFI were slightly below 0.90, they remained within the acceptable range for complex models. Meanwhile, CFI, TLI, and RMSEA achieved ideal thresholds, confirming that the measurement model demonstrated strong structural validity and internal consistency (Hair, 2009; Kline, 2023). These results provided a solid empirical foundation for subsequent chain mediation and path analyses, ensuring the reliability of inferential conclusions.

**TABLE 2:** Model fit indices.

Variable	Cronbach’s α	χ ^2^	*df*	χ ^2/^*df*	GFI	AGFI	TLI	CFI	RMSEA
Overall Model	0.957	1758.092	1366	1.287	0.887	0.878	0.979	0.980	0.024
AIPER	0.906	16.952	14	1.211	0.990	0.980	0.998	0.998	0.021
PU	0.959	49.405	20	2.47	0.976	0.956	0.990	0.993	0.055
PEOU	0.931	29.330	14	2.095	0.983	0.967	0.990	0.994	0.047
ATU	0.931	18.411	9	2.046	0.988	0.972	0.993	0.996	0.046
BI	0.855	6.463	5	1.293	0.995	0.984	0.997	0.998	0.024
HSE	0.935	26.096	20	1.305	0.987	0.976	0.997	0.998	0.025
SU	0.939	14.881	9	1.653	0.990	0.976	0.996	0.998	0.036
HBI	0.893	18.703	14	1.336	0.989	0.979	0.996	0.997	0.026

The criteria for model fit were: χ*^2/^df* < 3, GFI, AGFI, TLI, and CFI > 0.85, RMSEA < 0.06.

### 4.2 Confirmatory factor analysis

CFA was conducted for all observed indicators, and the covariances between latent and observed variables were examined. According to Brown (2015), standardized factor loadings above 0.50 are acceptable, while those above 0.70 are ideal. As shown in Table 3, the standardized loadings ranged from 0.742 to 0.969, indicating strong relationships between indicators and their corresponding latent constructs. The Composite Reliability (CR) values ranged from 0.896 to 0.966, demonstrating good internal consistency, while the Average Variance Extracted (AVE) values ranged from 0.610 to 0.780, suggesting that each construct explained over 60% of the variance in its indicators. In addition, Cronbach’s α coefficients ranged from 0.855 to 0.959, further supporting high reliability. Overall, all reliability and convergent validity indices met recommended standards: AVE values exceeded 0.50, CR values exceeded 0.70, and factor loadings exceeded 0.70, consistent with Fornell and Larcker (1981) and Hair (2009). These results indicate that the measurement model demonstrated strong reliability and validity, providing a solid basis for subsequent analyses.

**TABLE 3:** Confirmatory factor analysis.

Variable	Observed variable	Unstd	S.E	*Z*	*p*	Std.	SMC	CR	AVE	Cronbach’s α
*AIPER*	AIPER1	1				0.811	0.658	0.926	0.640	0.906
	AIPER2	0.956	0.055	17.511	***	0.798	0.637			
	AIPER3	0.879	0.052	16.791	***	0.779	0.607			
	AIPER4	0.934	0.055	17.078	***	0.785	0.616			
	AIPER5	1.008	0.055	18.160	***	0.817	0.667			
	AIPER6	0.950	0.055	17.164	***	0.789	0.623			
	AIPER7	1	0.055	18.233	***	0.820	0.672			
PU	PU1	1				0.969	0.939	0.966	0.780	0.959
	PU2	0.857	0.027	31.937	***	0.860	0.740			
	PU3	0.852	0.025	34.141	***	0.878	0.771			
	PU4	0.884	0.026	34.502	***	0.876	0.767			
	PU5	0.855	0.025	34.246	***	0.877	0.769			
	PU6	0.871	0.026	32.993	***	0.864	0.746			
	PU7	0.882	0.026	33.718	***	0.877	0.769			
	PU8	0.843	0.026	32.038	***	0.859	0.738			
PEOU	PEOU1	1				0.842	0.709	0.945	0.709	0.931
	PEOU2	0.991	0.048	20.524	***	0.836	0.699			
	PEOU3	1.016	0.047	21.553	***	0.859	0.738			
	PEOU4	0.964	0.048	20.219	***	0.834	0.696			
	PEOU5	1.040	0.048	21.741	***	0.863	0.745			
	PEOU6	0.966	0.047	20.517	***	0.834	0.696			
	PEOU7	0.953	0.047	20.151	***	0.826	0.682			
ATU	ATU1	1				0.867	0.752	0.945	0.742	0.931
	ATU2	1.031	0.043	23.866	***	0.881	0.776			
	ATU3	1.027	0.045	23.048	***	0.866	0.750			
	ATU4	1	0.044	22.793	***	0.864	0.746			
	ATU5	0.930	0.044	21.169	***	0.839	0.704			
	ATU6	0.968	0.044	22.025	***	0.852	0.726			
BI	BI1	1				0.800	0.640	0.896	0.634	0.855
	BI2	0.967	0.062	15.654	***	0.807	0.651			
	BI3	0.922	0.063	14.651	***	0.778	0.605			
	BI4	1.031	0.065	15.904	***	0.804	0.646			
	BI5	0.987	0.065	15.223	***	0.792	0.627			
HSE	HSE1	1				0.953	0.908	0.947	0.690	0.935
	HSE2	0.784	0.032	24.878	***	0.813	0.661			
	HSE3	0.823	0.031	26.556	***	0.817	0.667			
	HSE4	0.783	0.032	24.414	***	0.795	0.632			
	HSE5	0.810	0.032	25.226	***	0.812	0.659			
	HSE6	0.838	0.031	26.803	***	0.825	0.681			
	HSE7	0.796	0.031	25.443	***	0.811	0.658			
	HSE8	0.810	0.032	25.614	***	0.807	0.651			
SU	SU1	1				0.883	0.780	0.952	0.767	0.939
	SU2	0.975	0.041	23.557	***	0.870	0.757			
	SU3	0.913	0.039	23.292	***	0.864	0.746			
	SU4	0.988	0.041	24.219	***	0.879	0.773			
	SU5	0.978	0.040	24.481	***	0.881	0.776			
	SU6	0.977	0.041	24.092	***	0.878	0.771			
HBI	HBI1	1				0.799	0.638	0.916	0.610	0.893
	HBI2	0.950	0.060	15.779	***	0.766	0.587			
	HBI3	0.970	0.058	16.584	***	0.790	0.624			
	HBI4	1.014	0.060	16.821	***	0.797	0.635			
	HBI5	0.944	0.056	16.866	***	0.794	0.630			
	HBI6	0.966	0.060	16.173	***	0.776	0.602			
	HBI7	0.904	0.061	14.938	***	0.742	0.551			

***p < 0.001. T1: AIPER; PEOU; PU; ATU; BI; SU; HSE; T2: HBI.

### 4.3 Correlation analysis

As shown in Table 4, the descriptive statistical results indicate that the mean values (M) of the eight latent variables ranged from 4.064 to 5.113, suggesting that participants’ overall evaluations of each construct related to the AIPER and HBI were above the midpoint of the scale. The absolute values of skewness ranged from 0.067 to 0.520, and kurtosis ranged from 0.771 to 1.360, all within acceptable thresholds (| skewness| < 2, | kurtosis| < 8), indicating that the data distribution approximated normality.

**TABLE 4:** Descriptive statistics and correlation analysis of variables.

Variable	*M*	SD	Skew	Kurtosis	AVE	1	2	3	4	5	6	7	8
**1. AIPER**	4.593	4.593	−0.248	−1.292	0.640	***0.800***							
**2. PU**	4.887	4.887	−0.400	−1.255	0.780	0.439**	***0.883***						
**3. PEOU**	4.542	4.542	−0.246	−1.314	0.709	0.336**	0.426**	***0.842***					
**4. ATU**	4.454	4.454	−0.274	−1.127	0.742	0.457**	0.442**	0.462**	***0.861***				
**5. BI**	5.113	5.113	−0.442	−1.038	0.634	0.306**	0.377**	0.393**	0.425**	***0.796***			
**6. HSE**	4.064	4.064	−0.067	−1.360	0.690	0.348**	0.411**	0.414**	0.478**	0.321**	***0.831***		
**7. SU**	4.625	4.625	−0.404	−1.162	0.767	0.318**	0.415**	0.437**	0.465**	0.313**	0.421**	***0.876***	
**8. HBI**	5.024	5.024	−0.520	−0.771	0.610	0.135**	0.218**	0.136**	0.172**	0.129**	0.142**	0.417**	***0.781***

**p<0.01. The diagonal values in the correlation matrix represent the square roots of the AVE. Italic values on the diagonal represent the square roots of the Average Variance Extracted (AVE) for each construct. Off-diagonal values indicate inter-construct correlations. T1: AIPER, AI-based Personalized Exercise Recommendation System; PEOU, Perceived Ease of Use; PU, Perceived Usefulness; ATU, Attitude Toward Use; BI, Behavioral Intention; SU, System Use; HSE, Health Self-Efficacy; T2: HBI, Health Behavior Improvement.

Further analysis of the AVE across the eight latent variables revealed that the square root of each construct’s AVE exceeded its correlations with other constructs, confirming satisfactory discriminant validity among the variables. These findings demonstrate that the latent variables exhibited good internal differentiation and that the data met the requirements for subsequent structural modeling analysis.

### 4.4 Direct effects

According to the AMOS output (unstandardized coefficients), the path from the AIPER to PEOU was γ = 0.383, *p* < 0.001; the path from AIPER to PU was γ = 0.409, p < 0.001; PEOU → PU was γ = 0.408, *p* < 0.001; PU → ATU was γ = 0.187, *p* < 0.001; PEOU → HSE was γ = 0.293, *p* < 0.001; ATU → BI was γ = 0.416, *p* < 0.001; BI → SU was γ = 0.312, *p* < 0.001; HSE → SU was γ = 0.504, *p* < 0.001; HSE → HBI was γ = 0.336, *p* < 0.001; and SU → HBI was γ = 0.335, *p* < 0.001. These results indicate that PEOU, PU, ATU, BI, and SU all played significant roles in the process by which AIPER influenced HBI.

All direct paths reached statistical significance (*p* < 0.001), showing that the TAM structure remained stable and explanatory in the AI health-intervention context. In the extended model including HSE, the coefficients were PEOU → HSE: 0.293, *p* < 0.001; HSE → SU: 0.312, *p* < 0.001; and SU → HBI: 0.335, *p* < 0.001.

These findings suggest that HSE serves as an important psychological mediator in the chain from perceived ease of use to sustained health behavior improvement: when users perceive the system as easy to operate and providing clear feedback, their confidence in health management increases, which in turn raises system use and levels of health behavior improvement. Overall, the evidence supports H1–H6 and further shows that AIPER promotes HBI through dual cognitive and motivational pathways.

### 4.5 Chain mediation effects

**FIGURE 3:**
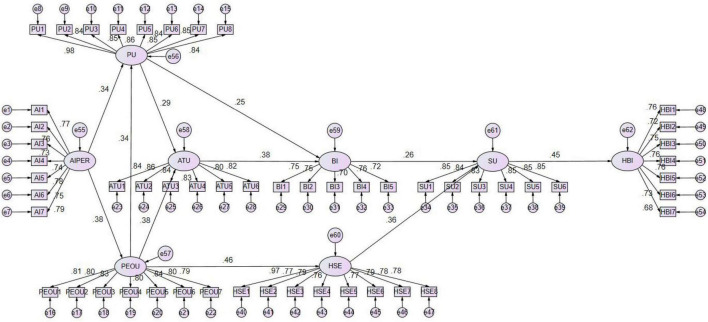
Chain mediation model. T1: AIPER; PEOU; PU; ATU; BI; SU; HSE. T2: HBI.

To enhance the accuracy of mediation testing, SEM was employed for indirect effect analysis, and the Bootstrap method was used to estimate the standard error of the chained mediation effect while further verifying its robustness. As shown in Figure 3 and Table 5, the total indirect effect of the AIPER on HBI was 0.0418 (SE = 0.0085, Z = Mean/SE), with an absolute Z value of 4.9647, exceeding the 1.96 threshold. The 95% confidence interval [0.0276, 0.0614] did not include zero (*p* < 0.001), indicating a statistically significant indirect effect.

*H1:*    In the process through which AIPER influenced HBI, the sequential pathway PEOU → PU → ATU → BI → SU exhibited a significant positive indirect effect (effect = 0.0013, 95% CI [0.0006, 0.0026], *p* < 0.001), confirming a stable chained mediation mechanism.
*H2:*    The pathway PEOU → PU → BI → SU also showed a significant positive indirect effect (effect = 0.0029, 95% CI [0.0013, 0.0062], *p* < 0.001), supporting the hypothesis of partial mediation.
*H3:*    The pathway PU → ATU → BI → SU demonstrated a significant positive indirect effect (effect = 0.0033, 95% CI [0.0016, 0.0067], *p* < 0.001), indicating that attitude and behavioral intention jointly mediated the relationship between PU and system use.
*H4:*    The pathway PU → BI → SU revealed a significant positive indirect effect (effect = 0.0077, 95% CI [0.0034, 0.0150], *p* < 0.001), showing that behavioral intention served as a key mediator between perceived usefulness and system use.
*H5:*    The full sequential pathway PEOU → PU → ATU → BI → SU → HBI had a significant chained mediation effect (effect = 0.0049, 95% CI [0.0021, 0.0095], *p* < 0.001), demonstrating that both cognitive and affective factors contributed to the sustained improvement of health behaviors among sedentary individuals.
*H6:*    The pathway PEOU → HSE → SU → HBI exhibited a significant positive indirect effect (effect = 0.0217, 95% CI [0.0129, 0.0353], *p* < 0.001), suggesting that HSE played a critical motivational role in linking system usability with long-term health behavior improvements.

**TABLE 5:** Chain mediation effects.

Hypothesis	Chained mediation	Estimate	Product of coefficients		Bias-corrected 95% CI		*p*
			S.E	Z	Lower	Upper	
H1	Effect “acdghk”	0.0013	0.0005	2.6000	0.0006	0.0026	0.0003
H2	Effect “acfhk”	0.0029	0.0012	2.5000	0.0013	0.0062	0.0006
H3	Effect “bdghk”	0.0033	0.0013	2.6154	0.0016	0.0067	0.0004
H4	Effect “bfhk”	0.0077	0.0029	2.7241	0.0034	0.0150	0.0008
H5	Effect “aeghk”	0.0049	0.0018	2.7222	0.0024	0.0099	0.0004
H6	Effect “aijk”	0.0217	0.0057	3.8070	0.0129	0.0358	0.0005
	Total	0.0418	0.0085	4.9647	0.0276	0.0614	0.0008

## 5 Discussion

This study extends the traditional Technology Acceptance Model (TAM) by uncovering differentiated psychological mechanisms through which AI-based personalized exercise recommendation systems promote health behavior improvement (HBI). The findings indicate that the influence of AIPER on HBI is not achieved through a single linear technological pathway, but rather through three distinct yet interrelated mechanisms—cognitive, affective, and motivational pathways. These results suggest that users not only need to cognitively perceive the system as easy to use and useful, but also need to develop positive emotional attitudes toward its use, and further build motivational beliefs that they are capable of adhering to AIPER-guided behaviors. Together, these mechanisms jointly drive sustained system use and eventually translate into improvements in health behaviors.

More importantly, these findings move beyond the mechanical assumption commonly seen in extended TAM studies that “more mediation paths imply stronger explanatory power.” Instead, this study provides a psychologically grounded explanation of how technology adoption is transformed into actual health behavior change in AI health-intervention contexts. Accordingly, the present study refines the theoretical interpretation of TAM, enriches its applicability to AI-driven health technology, and offers longitudinal empirical evidence and conceptual advancement for future research.

### 5.1 Cognitive mechanisms

The chained mediation pathway consisting of perceived ease of use (PEOU), perceived usefulness (PU), attitude toward use (ATU), behavioral intention (BI), and system use (SU) demonstrated a significant effect, supporting H1. For sedentary individuals, the main barrier to initiating behavior change is often not “lack of knowledge,” but “feeling troublesome.” When system registration, operation, and feedback are designed with low cognitive and operational burden, users experience reduced resistance and stronger willingness to explore. Consistent with TAM theory, PEOU alleviates psychological barriers by minimizing perceived effort, thereby enhancing PU (Davis, 1989; Venkatesh and Davis, 2000). Once users perceive small, immediate achievements such as successfully interrupting sedentary periods for several minutes, satisfaction and perceived control increase, which facilitates positive ATU, strengthens BI, and sustains SU.

Similarly, the cognitive pathway consisting of PEOU–PU–BI–SU (H2) was also supported. Compared with attitude-based mechanisms, this pathway operates through a more direct cognitive process. When AIPER functions effectively, provides clear structure, and reduces user uncertainty, PU strengthens rapidly and directly converts into BI (Venkatesh and Bala, 2008; Gefen and Straub, 2000). For sedentary users with limited discretionary time, “effectiveness” becomes a primary evaluation criterion; once usefulness is confirmed, users are more likely to initiate system use without requiring additional emotional reinforcement.

The PU–ATU–BI–SU pathway (H3) and PU–BI–SU pathway (H4) further reinforce the centrality of usefulness. When users believe that the AIPER meaningfully supports health goals, PU acts as the strongest determinant of ATU and BI (Chin et al., 2008; Rahimi et al., 2018). Positive attitudes reduce internal conflict (“knowing it is useful but not wanting to do it”), increasing behavioral determination and adherence. Additionally, continuous feedback, progress visualization, and performance tracking reinforce usefulness perceptions, forming a self-reinforcing cognitive loop and stabilizing long-term SU.

Beyond behavioral decision-making, growing evidence suggests that such sustained engagement with movement-related behaviors may further benefit cognitive functioning and emotional regulation. Zou and colleagues’ recent work on sedentary behavior and brain health indicates that interrupting prolonged sedentary patterns and engaging in regular physical activity is associated with improvements in cognitive processing efficiency, emotional stability, and overall brain health across the lifespan (Zou et al., 2024; Falck et al., 2025). In this regard, reductions in perceived effort and increases in perceived usefulness not only facilitate system acceptance, but may also indirectly create a psychologically and neurocognitively favorable condition for subsequent health behavior change.

### 5.2 Affective mechanisms

The pathway consisting of PEOU–ATU–BI–SU (H5) highlights an affective–experiential mechanism distinct from usefulness-driven mechanisms. When system interfaces are intuitive, responsive, and emotionally comfortable, users experience pleasure, fluency, and psychological comfort. These emotional responses convert into positive attitudes and promote proactive behavioral willingness rather than passive compliance (Tractinsky et al., 2000). Importantly, even when physiological benefits are not yet observable, enjoyable user experience and psychological comfort sustain early-stage engagement, thereby accelerating the transition from intention to behavior (Nov and Ye, 2008).

### 5.3 Motivational mechanisms

The PEOU–HSE–SU pathway (H6) demonstrates the motivational empowerment mechanism. When perceived operational burden is low, anticipated failure decreases, and users’ confidence in “being able to do it” increases (Fogg, 2009). Micro-task designs and frequent feedback cultivate mastery experiences, which are primary sources of self-efficacy (Bandura, 1997). Furthermore, adaptive rather than rigid recommendations help users maintain a sense of capability even when encountering setbacks, thereby preventing motivational collapse (Schwarzer, 2008). Compared with emotional attitudes or perceived usefulness, self-efficacy is more stable across time and contexts, providing a robust psychological foundation for persistent engagement and long-term behavioral improvement (McAuley and Blissmer, 2000).

From a design perspective, the HSE pathway suggests that AI-based exercise systems should move beyond simply improving ease of use and explicitly incorporate self-efficacy–enhancing features. In practice, this means providing graduated and achievable exercise goals, visualizing small but continuous progress, offering mastery-oriented feedback that emphasizes users’ capability (“you were able to complete a difficult session”), and including tolerance and recovery mechanisms for temporary lapses (e.g., plans for returning after inactivity) rather than penalizing failure. Such design decisions can gradually strengthen users’ health self-efficacy, which in turn supports long-term adherence and the transformation from intermittent system use to stable health behavior improvement.

### 5.4 From system use to health behavior improvement

Beyond traditional TAM studies that treat System Use (SU) as the terminal stage of behavioral response, the present study further links SU with Health Behavior Improvement (HBI). The findings indicate that sustained engagement with AIPER not only enhances exercise adherence, increases participation, and reduces prolonged sedentary behavior, but also gradually shapes healthier and more stable behavior patterns, thereby supporting positive HBI outcomes. In other words, SU is not merely a behavioral output of Behavioral Intention (BI); rather, it functions as a crucial mediating bridge connecting technology acceptance with tangible health consequences. This theoretical extension advances TAM from a framework centered primarily on “technology use” toward a more process-oriented model of “health effect realization.”

More importantly, this continuous transition from system use to health improvement is highly consistent with emerging sedentary behavior research. Existing evidence suggests that the health benefits of increased physical activity extend beyond physiological or metabolic domains to include enhanced cognitive functioning, improved emotional regulation, and better brain health across the lifespan. Reducing sedentary time and increasing active engagement have been shown to support neural functioning and psychological wellbeing in diverse populations (Zou et al., 2024). Therefore, within the context of AIPER, HBI should not be understood merely as behavioral optimization in terms of exercise frequency or regularity; instead, it represents a more comprehensive transformation that involves strengthened self-regulation capacity, improved cognitive efficiency, and increased emotional resilience.

### 5.5 Clarification of measurement nature and interpretation boundaries

Although this study reveals significant improvement in HBI associated with sustained AIPER use, it is important to note that HBI in this study reflects self-reported or perceived behavioral improvement, rather than directly measured behavioral or physiological indicators. Meanwhile, although a longitudinal design was adopted, the study remains observational rather than randomized experimental in nature. Therefore, the findings should be interpreted as model-consistent statistical associations, rather than definitive causal conclusions.

### 5.6 Comparison with existing TAM and mHealth evidence

Compared with existing TAM-based research in exercise technologies and mHealth, this study not only replicates the stable structural relationships among PU, PEOU, ATU, and BI, but also demonstrates more substantive enhancement in both effect magnitude and explanatory power. In the AI personalized exercise context, the BI→SU and SU?→HBI pathways exhibit stronger and more sustained influences, suggesting that adaptive and feedback-driven AI systems may more effectively translate cognitive evaluations into persistent behavior. Furthermore, by conceptualizing SU not as a terminal outcome but as a key mediating bridge toward HBI, and by incorporating Health Self-Efficacy (HSE) as an independent and essential motivational mechanism, the present model shows clearly improved explanatory capacity for behavioral persistence and long-term health improvement compared with traditional TAM frameworks. Although some prior studies have reported chain pathways linking cognition, intention, and system use, few have systematically revealed how cognitive, affective, and motivational mechanisms operate synergistically within a unified framework. Therefore, the contribution of this study lies in extending TAM beyond simple adoption outcomes by demonstrating, in a longitudinal AI exercise intervention context, a complete mechanism chain from “willingness to use,” to “ability to sustain use,” and finally to “actual health behavior improvement,” thereby providing deeper and theoretically stronger empirical evidence.

## 6 Limitations

Although the longitudinal SEM and Bootstrap analyses provide relatively strong support for the proposed psychological and behavioral mechanisms, the interpretation of “behavior change” in this study should still be treated with caution. First, the key behavioral indicators relied primarily on self-reported measures, which may be influenced by recall bias, social desirability, and subjective evaluation tendencies. Such self-reported outcomes may inflate the strength of mediation pathways—particularly those involving behavioral intention and health self-efficacy—making the associations appear stronger than they might be when assessed using objective behavioral indicators. Second, although common method bias was statistically examined and did not indicate a severe threat, the single-source and self-report nature of the data inevitably introduces a certain degree of shared method variance. Therefore, the findings of this study are more appropriately interpreted as theoretically meaningful, time-ordered associations rather than definitive causal evidence of health behavior change.

In addition, several methodological and external validity limitations remain. The sample was primarily drawn from specific populations (e.g., sedentary office workers), which restricts representativeness and generalizability; future research may expand to broader populations and contexts to enhance robustness. Moreover, although this study focused on the chain mediation mechanisms through which AI-based personalized exercise recommendations influence health behavior improvement, other potential mediating or moderating variables were not fully considered. Future work may incorporate additional contextual and individual-difference factors to develop a more comprehensive explanatory framework. It is also essential for subsequent studies to integrate objective behavioral indicators—such as device-based physical activity monitoring, system log data, and clinically assessed outcomes—to triangulate with self-reported data and further strengthen the objectivity, reliability, and external validity of the conclusions.

## 7 Conclusion

Based on a two-wave longitudinal dataset, this study extends the Technology Acceptance Model (TAM) by incorporating health self-efficacy and constructing a chained mechanism model of perception–attitude/intention–use-health behavior. The results demonstrate that AI-based personalized exercise recommendation systems can significantly enhance system use through multiple pathways involving perceived ease of use, perceived usefulness, attitude toward use, and behavioral intention, and further contribute to health behavior improvement among sedentary individuals. In particular, the pathway of perceived ease of use → health self-efficacy → system use reveals that interface and process optimization can be transformed into more robust capability beliefs and sustained behavioral engagement.

From a practical perspective, the findings suggest that AI-based personalized exercise recommendation systems may serve as a valuable tool to support health behavior improvement in sedentary populations. Although this study is not a randomized intervention trial, the observed associations indicate that system use of such systems may be associated with enhanced motivation, improved adherence, and more stable engagement in health-related behaviors. Therefore, relevant institutions and policymakers may consider incorporating such systems as complementary components within broader health promotion strategies, while developers may prioritize enhancing usability, functionality, emotional experience, and self-efficacy–supportive features. At the policy level, such systems have potential application value in community health promotion, workplace sedentary behavior management, and preventive health programs; however, their large-scale implementation should be approached cautiously, with attention to data security, privacy protection, accessibility, and equity, and still requires further experimental and longitudinal evidence to confirm causal effects and cross-context applicability.

Compared with existing TAM-based research in the field of digital health, this study provides several important theoretical extensions. First, moving beyond the predominance of cross-sectional designs, it adopts a longitudinal approach to examine the dynamic association between system use and health behavior improvement, offering time-based evidence for understanding behavior change processes. Second, by integrating health self-efficacy into the extended TAM framework, this study not only considers technological characteristics and usage intention, but also highlights the critical role of individual psychological resources in the transition from “technology adoption” to “behavioral change.” Finally, by linking system use with health behavior improvement, the study extends the explanatory focus from “tool usage” to “health-related behavioral outcomes,” thereby providing a more coherent theoretical pathway for understanding how digital health tools contribute to real behavioral change. Collectively, these contributions indicate that this study is not a simple replication of TAM, but a contextually meaningful deepening and extension of TAM within a health intervention setting.

## Data Availability

The datasets presented in this study can be found in online repositories. The names of the repository/repositories and accession number(s) can be found at: https://doi.org/10.6084/m9.figshare.30513980.
